# Supplementary figures and images for: Lack of Adiponectin Drives Hyperosteoclastogenesis in Lipoatrophic Mice
Source: Front Cell Dev Biol. 2021 Apr 1;9:627153. doi: 10.3389/fcell.2021.627153 (PMC8047205; doi:10.3389/fcell.2021.627153)

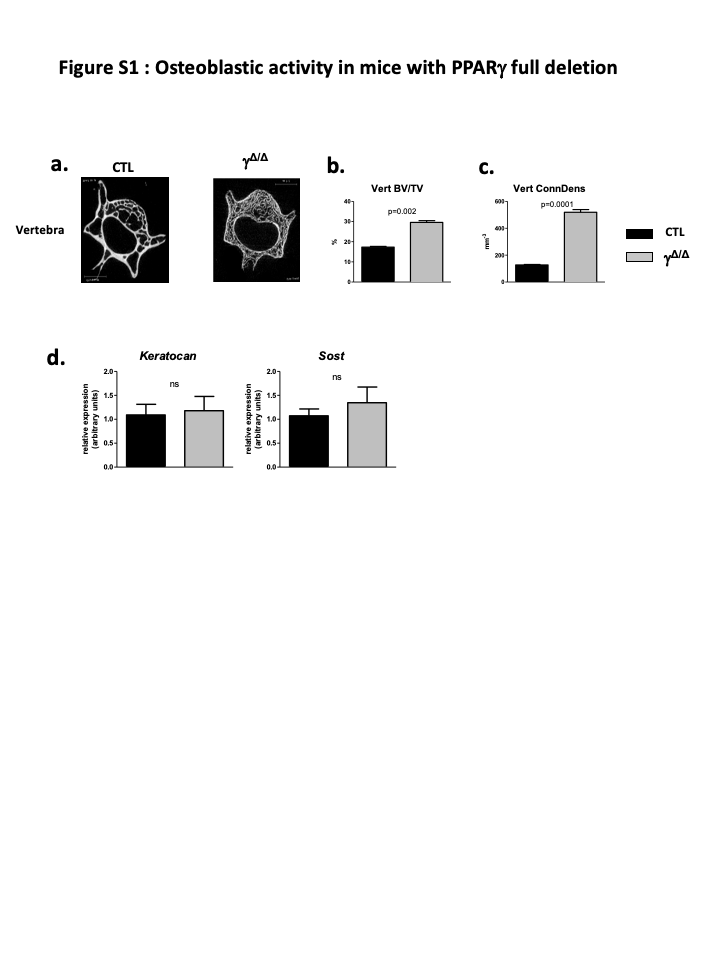

Supplement: Supplementary Figure 1 — Osteoblastic activity is increased in mice with epiblastic deletion of PPARg. (A) Representative 3-D micro-CT of the fourth lumbar vertebrae of CTL and PpargΔ/Δ (γΔ/Δ) mice. n = 5 WT and 5 γΔ/Δ. (B) Trabecular bone volume fraction measured by micro-CT analysis of vertebra from γΔ/Δ (n = 5) and control (CTL; n = 5) littermates. (C) Trabecular connectivity density measured by micro-CT analysis of vertebra from γΔ/Δ (n = 5) and control (CTL; n = 5) littermates. (D) Keratocan and Sost mRNA levels in long bones of CTL (n = 6) and γΔ/Δ (n = 6) mice. [file Image_1.TIFF]

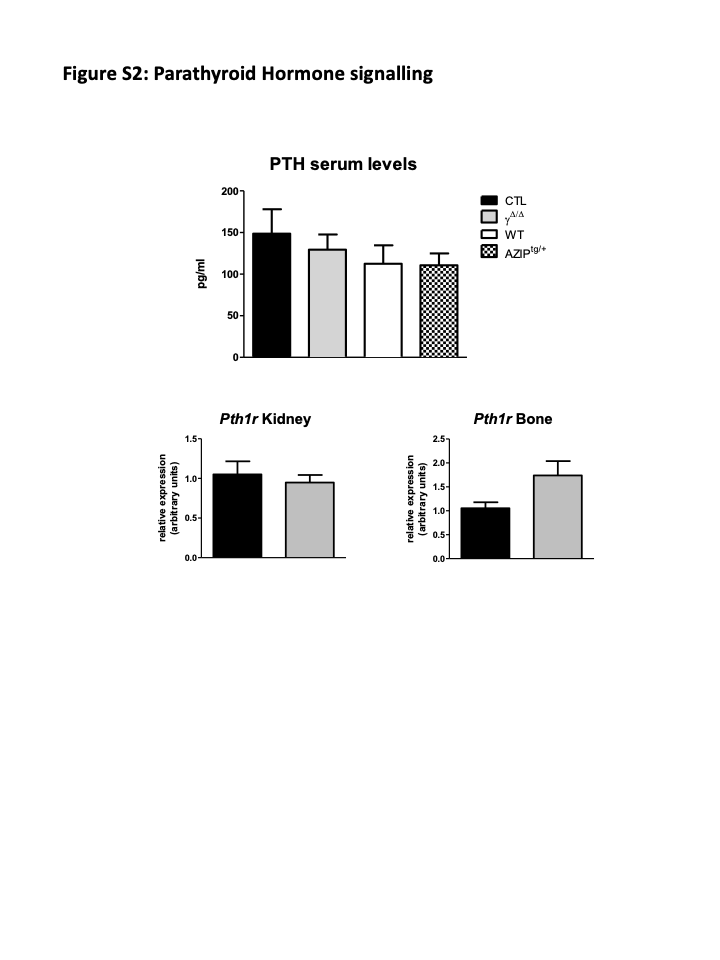

Supplement: Supplementary Figure 2 — Parathyroid hormone (PTH) signaling. The serum levels of PTH were assessed by Elisa Assay in 1-year-old male and female mice (PpargΔ/Δ, AZIPtg/+, and their control littermates). Six males and six females were included per genotype. As there were no statistically significant differences between males and females, the analysis was performed using the total number of mice (mean ± SD). Lower panels: mRNA expression levels of Pth1r in the bones and in the kidneys of CTL vs. PpargΔ/Δ mice. [file Image_2.TIFF]

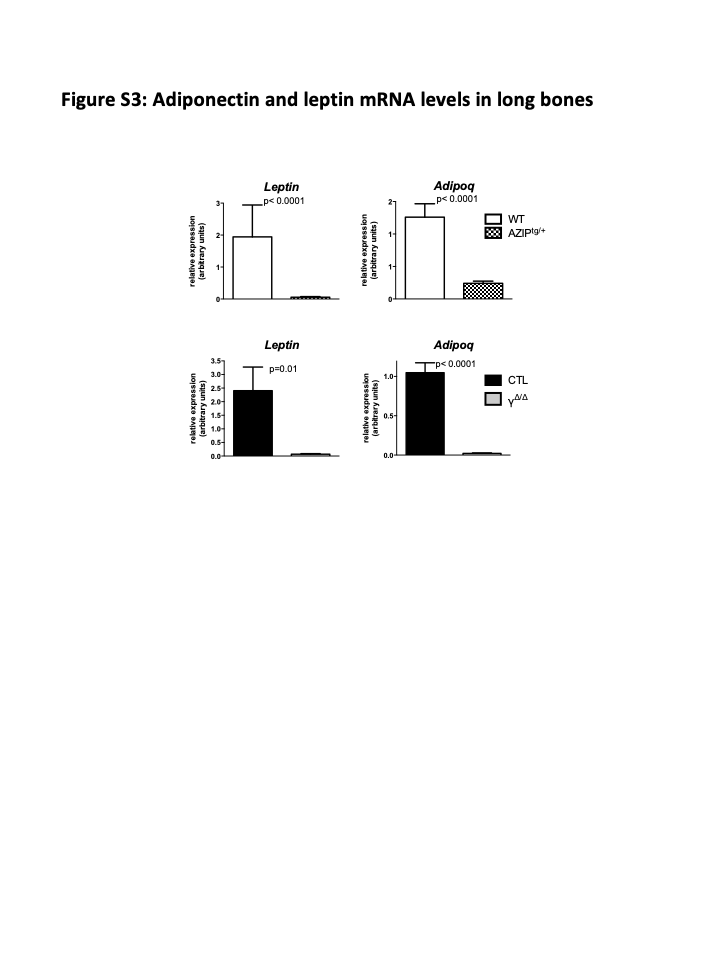

Supplement: Supplementary Figure 3 — Adiponectin expression levels in long bone and in adipocyte-conditioned medium. RT-qPCR analysis of Leptin and AdipoQ mRNA levels in long bone from A-ZIPtg/+ (n = 8) and their CTL littermates (n = 8) and from γΔ/Δ (n = 7) and their CTL littermates (n = 6). Data are presented as mean ± S.E.M. Statistical significance was determined by two-tailed unpaired t-test. [file Image_3.TIFF]

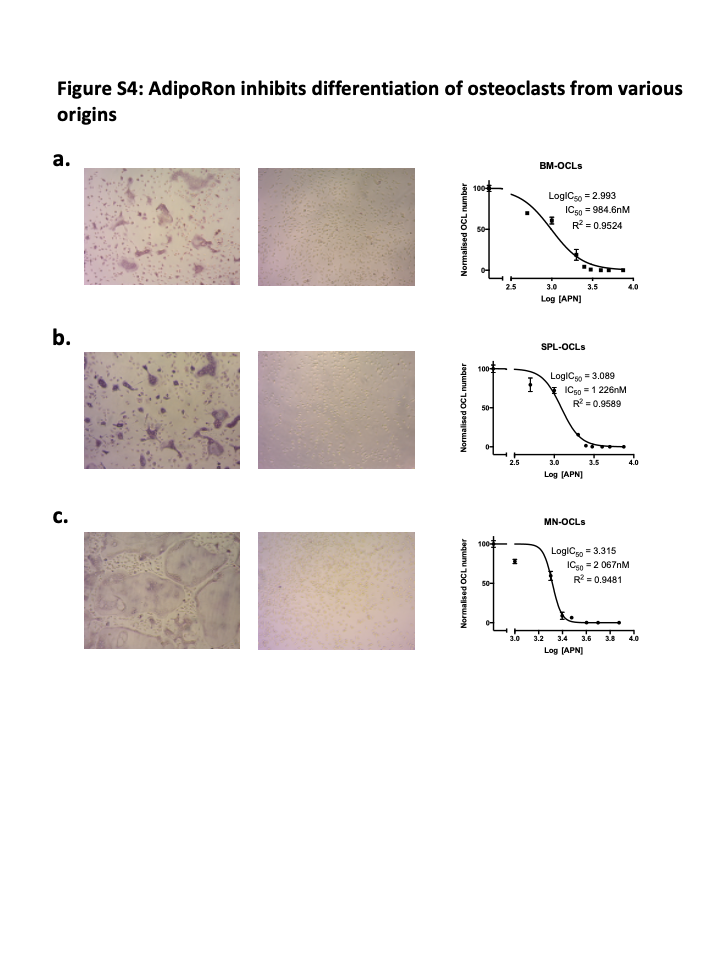

Supplement: Supplementary Figure 4 — AdipoRon inhibits osteoclast (OCL) differentiation of various origins. Left panels: representative images of TRAcP stained (A) BM-derived, (B) spleen derived, and (C) macrophages enriched fraction osteoclasts differentiated in the presence or absence of AdipoRon (5 μM). Right panels: the mean IC50 of AdipoRon inhibition of OCL differentiation was determined from the curve with the error of the fit (S.E.M.) Data are mean ± S.E.M. (n = 3 biological replicates). [file Image_4.TIFF]

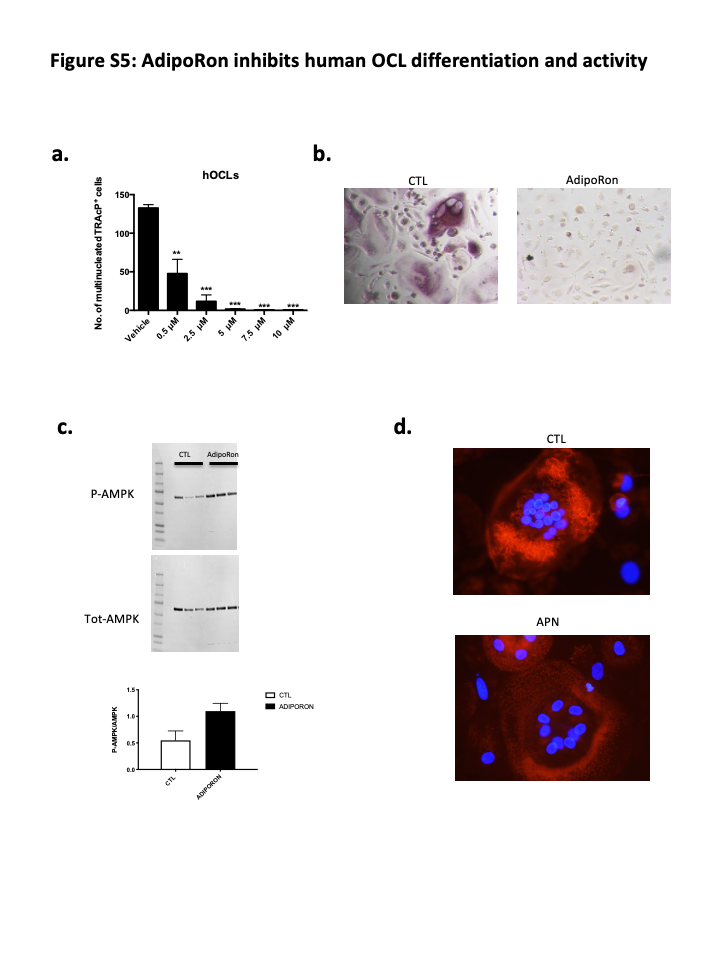

Supplement: Supplementary Figure 5 — AdipoRon inhibits human osteoclast (OCL) differentiation and activity through AMPK activation. (A) Dose response effect of AdipoRon on osteoclast differentiation from hPBMC (n = 5). (B) Representative images of TRAcP staining of hPBMC derived osteoclast differentiation in the presence or absence of AdipoRon (5(μM). (C) Western-blot analysis of hPBMC-derived osteoclasts treated with AdipoRon (5(μM). AdipoRon treatment activates AMPK phosphorylation (p-AMPK) in hPBMC after 5 min. (D) Fluorescence microscopy of phalloidin-marked podosomes in hPBMC derived OCL in the presence of AdipoRon (5 μM, 12 h) (representative images of n = 5). [file Image_5.TIFF]
