# Supplementary material for: Lack of Adiponectin Drives Hyperosteoclastogenesis in Lipoatrophic Mice
Source: Front Cell Dev Biol. 2021 Apr 1;9:627153. doi: 10.3389/fcell.2021.627153 (PMC8047205; doi:10.3389/fcell.2021.627153)
Supplement: Supplementary Table 1 — Primers used for q-RT-PCR. [file Table_1.docx]

**Supplementary Table 1: Primers used for q-RT-PCR.**

| **Gene** | **Fwd** | **Rev** |
| --- | --- | --- |
| ALP | AAC CCA GAC ACA AGC ATT CC | CCA GCA AGA AGA AGC CTT TG |
| OC | TTC TGC TCA CTC TGC TGA CC | TTT GTA GGC GGT CTT CAA GC |
| Pparγ | AAG AGC TGA CCC AAT GGT TG | GCA TCC TTC ACA AGC ATG AA |
| Acp5 | TGC CTA CCT GTG TGG ACA TGA | CAC ATA GCC CAC ACC GTT CTC |
| MMP9 | CTC ATG TAC CCG CTG TAT AG | TTA GAG CCA CGA CCA TAC A |
| Ctsk | CAG CAG AGG TGT GTA CTA TG | GCG TTG TTC TTA TTC CGA GC |
| Keratocan | GCA GCA CCT TCA CCT TGA TC | AGG TAG CTC AGT TGT GGT CC |
| Sost | ACA ACC AGA CCA TGA ACC G | CAG GAA GCG GGT GTA GTG |
| Leptin | GTG CCT ATC CAG AAA GTC CAG | TGA AGC CCA GGA ATG AAG TC |
| AdipoQ | AGG CAT CCC AGG ACA TC | CCT GTC ATT CCA ACA TCT CC |
| AdipoR1 | TTT GCC ACT CCC AAG CAC | ACA CCA CTC AAG CCA AGT CC |
| AdipoR2 | TCT CAG TGG GAC ATG TTT GC | AGG CCT AAG CCC ACG AAC |
| Pth1R | CCC CGA GTC TAA AGA GAA CAA G | GTA ATC GGG ACA AGG TAC TGC |
| Gapdh | TTG ATG GCA ACA ATC TCC AC | CGT CCC GTA GAC AAA ATG GT |
| Actb | CTA AGG CCA ACC GTG AAA AGA T | CAC AGC CTG GAT GGC TAC GT |
| 36B4 | CGA CCT GGA AGT CCA ACT AC | ATC TGC TGC ATC TGC TTG |
